# Supplementary material for: Early life stress alters transcriptomic patterning across reward circuitry in male and female mice
Source: Nat Commun. 2019 Nov 8;10:5098. doi: 10.1038/s41467-019-13085-6 (PMC6841985; doi:10.1038/s41467-019-13085-6)
Supplement: Supplementary file 1 — Supplementary Information [file 41467_2019_13085_MOESM1_ESM.pdf]

Supplementary Information

Peña et al., **Early life stress alters transcriptomic patterning across reward circuitry in male and female mice**

Supplementary Figure 1

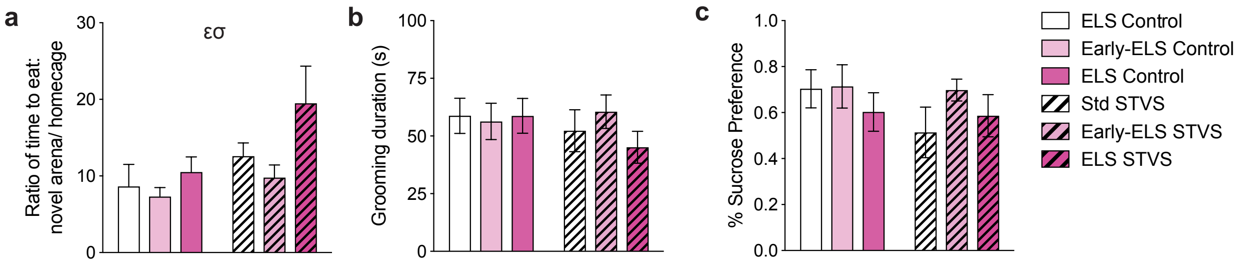

Supplementary Figure 1 | Early-ELS does not significantly alter depression-like behavior before or after STVS.

a, Ratio of latency to eat in a novel arena vs home cage in the novelty suppressed feeding test in a behavioral replication cohort of adult female mice. There was not a main effect of cohort (ANOVA: p=0.723) and combined cohorts yielded a significant interaction between ELS and STVS (ANOVA: $F_{1,81}$=5.326, p=0.024), main effect of ELS ($F_{1,81}$=11.225, p=0.001) and main effect of STVS ($F_{1,81}$=10.376, p=0.002) on NSF ratio. **b**, Grooming duration in the splash test: within the replication cohort alone there was no main effect of either stress on splash test grooming duration. There was not a main effect of cohort (p=0. 800) and combined cohorts yielded trends for main effects of ELS ($F_{1,78}$=3.746, p=0.057) and STVS ($F_{1,78}$=2.906, p=0.092). **c**, Sucrose preference in a two-bottle choice test: while there was again a lack of effect of ELS or STVS on sucrose preference in females in the replication cohort, when cohorts were combined there was an interaction trend ($F_{1,81}$=2.928, p=0.091). Significant (p<0.05) main effect of ELS (ε) or of adult stress (σ) as indicated. Error bars indicate mean ± SEM.

Supplementary Figure 2

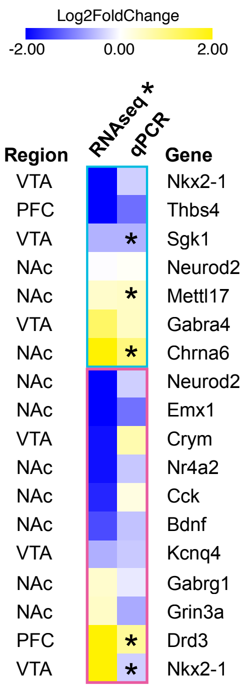

Supplementary Figure 2 | Gene expression changes from independent cohorts of mice.
Expression change for a semi-random subset of genes was analyzed by qPCR for the ELS vs Std comparison, including genes from each region and sex. Validation tissue was collected from cohorts completely independent from the original RNA-seq samples. ELS-induced expression change is represented by Log2(fold change) from Std and compared to original change found by RNA-seq. Genes are separated by sex (as indicated by blue and pink boxes) and sorted by magnitude of change in RNA-seq. * $p<0.1$ by two-tailed t-test.
