# Supplementary material for: Early life stress alters transcriptomic patterning across reward circuitry in male and female mice
Source: Nat Commun. 2019 Nov 8;10:5098. doi: 10.1038/s41467-019-13085-6 (PMC6841985; doi:10.1038/s41467-019-13085-6)
Supplement: Supplementary file 3 — Description of Additional Supplementary Files [file 41467_2019_13085_MOESM3_ESM.pdf]

Description of Additional Supplementary Files

File Name: Supplementary Data 1
Description: RNA-seq mapping rate

File Name: Supplementary Data 2
Description: Differentially expressed genes at >30% fold-change and $p<0.05$

File Name: Supplementary Data 3
Description: Primed genes

File Name: Supplementary Data 4
Description: qPCR Primers
