# Supplementary material for: Early life stress alters transcriptomic patterning across reward circuitry in male and female mice
Source: Nat Commun. 2019 Nov 8;10:5098. doi: 10.1038/s41467-019-13085-6 (PMC6841985; doi:10.1038/s41467-019-13085-6)
Supplement: Supplementary file 8 — Reporting Summary [file 41467_2019_13085_MOESM8_ESM.pdf]

nature research

Corresponding author(s): Catherine J Peña; NCOMMS-19-13687

Last updated by author(s): Sep 25, 2019

Reporting Summary

Nature Research wishes to improve the reproducibility of the work that we publish. This form provides structure for consistency and transparency in reporting. For further information on Nature Research policies, see Authors & Referees and the Editorial Policy Checklist.

Statistics

For all statistical analyses, confirm that the following items are present in the figure legend, table legend, main text, or Methods section.

n/a	Confirmed	
☐	☒	The exact sample size (*n*) for each experimental group/condition, given as a discrete number and unit of measurement
☐	☒	A statement on whether measurements were taken from distinct samples or whether the same sample was measured repeatedly
☐	☒	The statistical test(s) used AND whether they are one- or two-sided<br>*Only common tests should be described solely by name; describe more complex techniques in the Methods section.*
☐	☒	A description of all covariates tested
☐	☒	A description of any assumptions or corrections, such as tests of normality and adjustment for multiple comparisons
☐	☒	A full description of the statistical parameters including central tendency (e.g. means) or other basic estimates (e.g. regression coefficient) AND variation (e.g. standard deviation) or associated estimates of uncertainty (e.g. confidence intervals)
☐	☒	For null hypothesis testing, the test statistic (e.g. *F*, *t*, *r*) with confidence intervals, effect sizes, degrees of freedom and *P* value noted<br>*Give P values as exact values whenever suitable.*
☒	☐	For Bayesian analysis, information on the choice of priors and Markov chain Monte Carlo settings
☐	☒	For hierarchical and complex designs, identification of the appropriate level for tests and full reporting of outcomes
☒	☐	Estimates of effect sizes (e.g. Cohen's *d*, Pearson's *r*), indicating how they were calculated

Our web collection on statistics for biologists contains articles on many of the points above.

Software and code

Policy information about availability of computer code

Data collection	Behavioral data was collected with Ethovision (Noldus) and by hand into Excel spreadsheets. All behavior (Ethovision + hand-scored) was organized in Excel before analysis.
Data analysis	Statistical analyses of behavior were done with SPSS (IBM, v25) and Prism (GraphPad, v8) as indicated.<br>RNAseq analysis used pipelines available and described in detail online, as cited, including: Tophat2, HTSeq-counts, and DESeq2 (all in R), Morpheus (online free software), GeneOverlap (R tool), DAVID (online free tool), HOMER (in R), Ingenuity Pathway Analysis (Qiagen, proprietary software), Rank-rank hypergeometric overlap analysis (R tool), Plasticity signature analysis as described in Smith et al. (eNeuro, 2016; PMCID: PMC5241709).

For manuscripts utilizing custom algorithms or software that are central to the research but not yet described in published literature, software must be made available to editors/reviewers. We strongly encourage code deposition in a community repository (e.g. GitHub). See the Nature Research guidelines for submitting code & software for further information.

Data

Policy information about availability of data

All manuscripts must include a data availability statement. This statement should provide the following information, where applicable:

- Accession codes, unique identifiers, or web links for publicly available datasets
- A list of figures that have associated raw data
- A description of any restrictions on data availability

All RNAseq data generated and analyzed in this mamuscript are available online through GEO, accession GSE89692.
All behavioral data is shown in individual data points in Figure 1, included in a Source Data file, or may be requested from corresponding author CJP at cpena@princeton.edu.

Field-specific reporting

Please select the one below that is the best fit for your research. If you are not sure, read the appropriate sections before making your selection.

☒ Life sciences ☐ Behavioural & social sciences ☐ Ecological, evolutionary & environmental sciences

For a reference copy of the document with all sections, see nature.com/documents/nr-reporting-summary-flat.pdf

Life sciences study design

All studies must disclose on these points even when the disclosure is negative.

Sample size	Power analysis for numbers of female mice used in behavioral tests was based on variability from our previous male study of early life stress, and indicated we should use ~13 mice/group. We strove to include 13 mice/ group in our initial behavioral cohort, and ended up ranging from 12-15 based on numbers of successful litters.<br>Sample size for RNAseq ranged from 4-8 per group. Standard-reared and ELS control (no adult stress) male VTA and NAc RNAseq datasets had only 4 samples/group (from 2-3 pooled mice/sample), while all other male groups and all female groups had 6-8 samples/group (with samples representing individual mice). These numbers were based on RNA quality and expense of sequencing, with greater numbers of samples included as sequencing prices came down.
Data exclusions	As described, outliers in behavioral data were calculated as greater than 2 standard-deviations from the group mean, and were excluded. These criteria were set prior to analysis and were evenly applied across all groups and analyses.<br>RNAseq outliers were determined by PCA analysis, but none were detected and all samples were included in analysis.
Replication	We attempted to replicate behavioral results in a second, independent cohort of animals, including a third early life manipulation ("early-ELS"). Sample numbers ranged from 9-21, with more mice allotted towards the novel group and depending on breeding success. Discrepancies between initial cohort (Fig 1) and replication cohort (Supplemental Fig. 1) are described in the main text results. Cohort was used as a factor, and when not significant, results were reported for the combined cohorts, which predominately upheld the behavioral findings of the initial cohort.
Randomization	Litters were randomly assigned to early life conditions (Std vs ELS, or early-ELS in the replication cohort). Half of each litter was then randomly assigned to each adult condition (Control or STVS). Male behavior, while reported previously, was randomized identically (Peña et al., Science, 2017). Samples for RNAseq were selected across the range of behaviors of each group in order to represent the full group (including both susceptible and resilient, in proportion to the group)- based primarily on novelty suppressed feeding outcomes for females, and on social interaction behavior for males.
Blinding	Analysis of behavioral data was done with observers blind to condition.

Reporting for specific materials, systems and methods

We require information from authors about some types of materials, experimental systems and methods used in many studies. Here, indicate whether each material, system or method listed is relevant to your study. If you are not sure if a list item applies to your research, read the appropriate section before selecting a response.

Materials & experimental systems

n/a	Involved in the study	
☒	☐	Antibodies
☒	☐	Eukaryotic cell lines
☒	☐	Palaeontology
☐	☒	Animals and other organisms
☒	☐	Human research participants
☒	☐	Clinical data

Methods

n/a	Involved in the study	
☒	☐	ChIP-seq
☒	☐	Flow cytometry
☒	☐	MRI-based neuroimaging

Animals and other organisms

Policy information about studies involving animals; ARRIVE guidelines recommended for reporting animal research

Laboratory animals	All mice were wildtype C57Bl/6J mice. Nulliparous breeders were ordered from Jackson as adults and mated in our facilities. ELS was performed as described either from postnatal day 10-17 or P2-10. Behavioral testing took place from P60-80. Tissue was collected on the day following final behavioral testing. Female behavior is reported here for the first time. RNAseq was performed using both male and female tissue (with male behavior previously reported: Peña et al., Science, 2017).
Wild animals	N/A
Field-collected samples	N/A

Ethics oversight

All experiments were conducted in accordance with the guidelines of the Institutional Animal Care and Use Committee at Mount Sinai and of the Society for Neuroscience.

Note that full information on the approval of the study protocol must also be provided in the manuscript.
